# Correction: A possible new mechanism and drug intervention for kidney damage due to arsenic poisoning in rats

**DOI:** 10.1039/c6tx90006b

**Published:** 2016-02-24

**Authors:** Yu-yan Xu, Qi-bing Zeng, Mao-lin Yao, Chun Yu, Jun Li, Ai-hua Zhang

**Affiliations:** a Key Laboratory of Environment Pollution Monitoring and Disease Control , Ministry of Education , Department of Toxicology , School of Public Health , Guizhou Medical University , Guiyang , Guizhou 550025 , China . Email: aihuagzykd@163.com ; Fax: +86851 8841 6175 ; Tel: +86 851 8841 6172

## Abstract

Correction for ‘A possible new mechanism and drug intervention for kidney damage due to arsenic poisoning in rats’ by Yu-yan Xu, *et al.*, *Toxicol. Res.*, 2016, DOI: 10.1039/c5tx00165j.



## 

The Acknowledgements section should read:

This work was supported by the National Natural Science Foundation of China (no. 81430077, 81172603).

The Royal Society of Chemistry apologises for these errors and any consequent inconvenience to authors and readers.

